# Application of fingernail samples as a biomarker for human exposure to arsenic-contaminated drinking waters

**DOI:** 10.1038/s41598-022-08845-2

**Published:** 2022-03-18

**Authors:** Reza Shokoohi, Mohammad Khazaei, Manoochehr Karami, Abdolmotaleb Seid-mohammadi, Salman Khazaei, Zahra Torkshavand

**Affiliations:** 1 Department of Environmental Health Engineering, School of Public Health and Research Center for Health Sciences, Hamadan University of Medical Sciences, Hamadan, Iran; 2 Department of Epidemiology, School of Public Health and Safety, Shahid Beheshti University of Medical Sciences, Tehran, Iran; 3 Research Center for Health Sciences, Hamadan University of Medical Sciences, Hamadan, Iran

**Keywords:** Environmental sciences, Natural hazards, Biomarkers, Risk factors

## Abstract

This study evaluated the relationship between arsenic uptake via drinking water ingestion and arsenic concentration in fingernails as a biomarker for human exposure. For this purpose, we collected fingernail samples from 40 healthy participants of arsenic-affected rural regions of Kaboudrahang County, the west of Iran. A total of 49 fingernail samples were also collected from individuals who lived in areas where contamination of drinking water sources with arsenic had not been reported. It was found that the fingernails arsenic contents in 50 and 4.08% of the samples collected from arsenic-contaminated and reference villages were higher than the normal arsenic values of nails (0.43–1.08 µg/g), respectively. Based on the results of adjusted multiple linear regression, a significant association was found between groundwater and fingernails arsenic concentration (*p* < 0.001). Moreover, a statistically significant association was shown between arsenic in the fingernail samples and gender (*p* = 0.037). Fingernails arsenic contents were not significantly affected by other variables including age, smoking habits, and BMI (*p* > 0.05). In light of the results of this study, the use of biological indicators such as fingernail tissues due to easier sampling and less risk of external contamination is suitable for assessing exposure to heavy metals in contaminated areas.

## Introduction

Arsenic is considered one of the dangerous metalloids occurring in drinking water sources through natural and anthropogenic activities. Anthropogenic activities such as the application of herbicides, pesticides, wood timber preservatives, and metal smelting industries, and natural sources of arsenic exposure including geothermal processes, volcanic eruptions, and weathering of mineral lead to widespread arsenic contamination in subsurface environments like sediment, soil, surface water, and groundwater sources^1–5^. This metalloid recognized as a human carcinogen by the International Agency for Research on Cancer (IARC) and the United States Environmental Protection Agency (USEPA) is found in organic and inorganic forms and with different oxidation states (− 3, 0, + 3, + 5) in the environment^6,7^. Non-occupational exposure to inorganic arsenic (arsenite and arsenate) through the consumption of drinking water sources causes carcinogenic and non-carcinogenic health effects in the exposed human population^3,8,9^. Acute and chronic human health effects of arsenic exposure include skin lesions, cardiovascular disease, anemia, renal impairment, as well as respiratory disorders, and different types of cancers (lung, skin, liver, kidney, and bladder)^10–12^. Therefore, to ensure the health of water for drinking purposes, the maximum allowable level for arsenic has recommended by the World Health Organization is 10 µg/L^13,14^. In addition, due to the widespread occurrence of arsenic in the environment and its potential for adverse effects on human health, biological monitoring of pollutants is of great importance in toxicological studies for assessing human exposure through natural and anthropogenic sources. Biological monitoring usually involves collecting fluids and tissues from the human body and analyzing them to identify the chemicals or their metabolites^15,16^. There are several biomarkers to identify and evaluate human exposure monitoring to arsenic and its compounds. Blood, urine, hair, and nails have been identified as easily accessible biomarkers to assess arsenic exposure in epidemiological studies^17^. Among these bioindicators, the amount of arsenic measured in blood and urine samples indicates recent intake of arsenic, on the order of approximately four days in the urine samples and 2–6 h in the blood samples^18^. On the other hand, the need to freeze collected urine samples, the invasive nature of blood sample collection methods, and the storage issues associated with these samples are significant drawbacks related to the use of these biomarkers^19^. While the amount of arsenic measured in hair and nail samples reflect exposure during a longer period (from 3 to 6 months prior) compared to body fluids. For this reason, in populations that are exposed to high levels of arsenic for a long time, especially through the ingestion of drinking water, these biomarkers (hair and nail samples) are used to quantify levels of exposure^18,20^. It should be noted that most of the consumed arsenic through drinking water excreted as methylated arsenic within 1–3 days following exposure. However, some of the absorbed inorganic arsenic into the body has a high affinity to bind to sulfhydryl-groups and accumulate in keratin-dense tissues like nails and hair^18,21^. The use of hair samples as a biomarker has been discussed due to the highly variable growth rate, the need to know the biology of hair, and the possibility of external contamination compared to nail samples. While the use of nail samples as bioindicators has more advantages due to less possibility of external contamination and much slower growth rate (0.9–1.5 mm/month) compared to the hair samples (6–36 mm/month)^17^. Therefore, this study aimed to evaluate the association between arsenic exposure from ingestion of drinking water and fingernail arsenic levels among the individuals who lived in the contaminated rural areas of Kaboudrahang County in the west of Iran, where exposed to arsenic concentrations in groundwater as the main source of drinking water ranging from very low to high content.

## Methods

### Description of the study area and population

**Figure 1:**
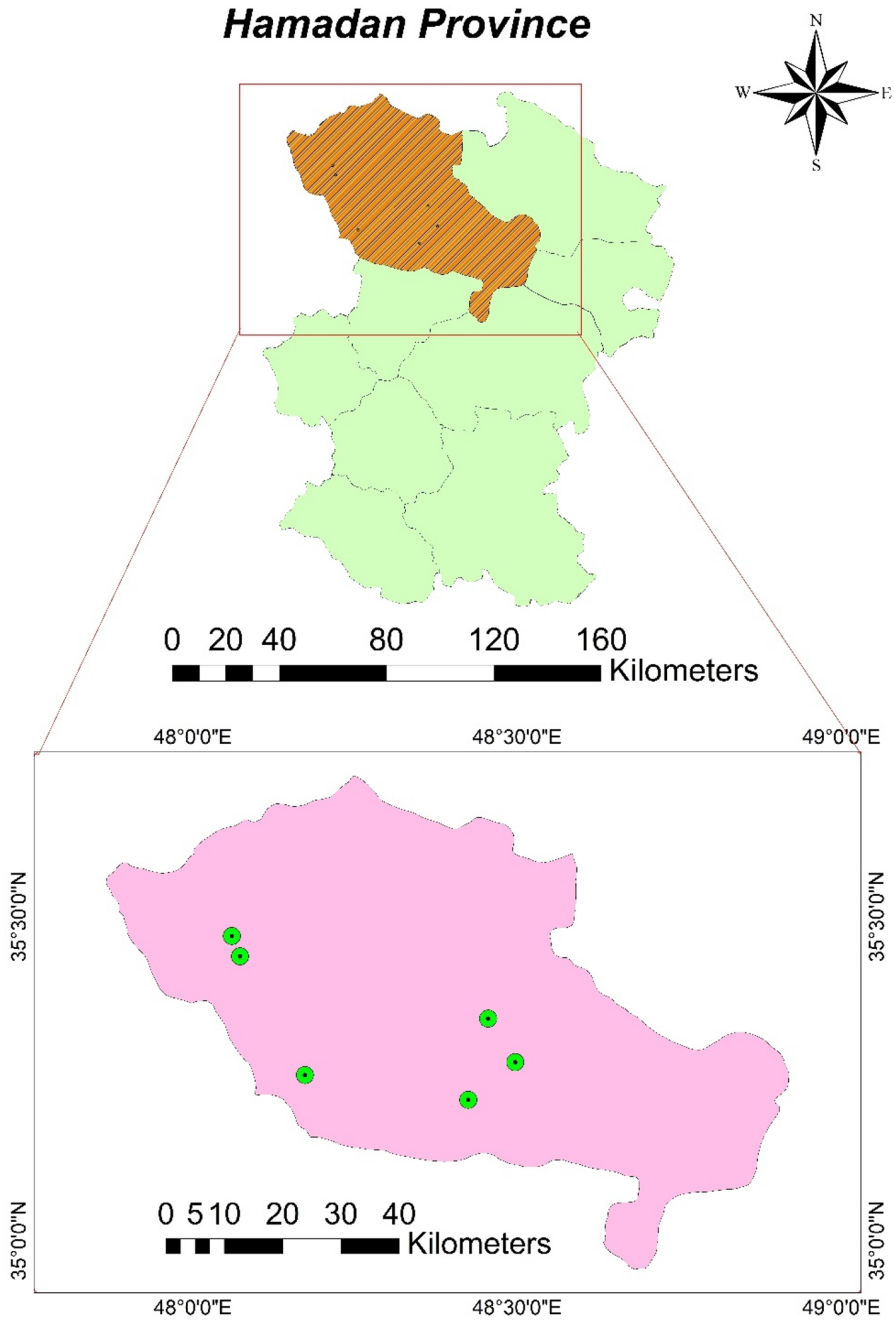
Location of the study area and selected village for fingernails sampling (ArcGIS version 10.4.1).

In this cross-sectional study, the fingernail samples were collected from 89 healthy participants living in rural areas located in the west of Kaboudrahang County, Hamadan Province, Iran. The location of the villages selected for the fingernail sampling of the participants in the area studied has been shown in Fig. 1. It should be noted that the location of the study area and selected villages for fingernail sampling were attained using ArcGIS version 10.4.1 (Fig. 1). The levels of arsenic in groundwater sources were used as a basis for the selection of villages for the fingernail sampling of participants. Thus, three arsenic-affected villages were considered as the exposed group and three villages with lower concentrations of arsenic were considered as the reference group. In these regions, groundwater sources are considered as the main supply of water for agriculture and drinking demands. It is worth noting that all the individuals in the current investigation were participants who lived in rural regions and had no occupational exposure to arsenic. Also, the local economy of the selected areas is mostly based on agriculture and animal husbandry. Then, information such as gender, height, weight, age, and smoking was also obtained from all participants using a self-reported questionnaire. Body Mass Index (BMI) was calculated as weight (kilograms) divided by height (meters squared). The study protocol was reviewed and approved by the ethical committee of Hamadan University of Medical Sciences (approval number IR. UMSHA.REC.1397.916) and all experiments were performed by relevant guidelines and regulations. For ethical permission before filling out the questionnaires, informed consent was obtained from all participants and/or their legal guardians. Finally, all the participants were assured that their information would remain confidential.

### Sample collection

A total of six groundwater samples were collected from the public water distribution system network of each village in the area studied in polyethylene bottles for arsenic measurement, followed by the addition of one drop of 65% HNO_3_. Then, the acidified groundwater samples were kept in the refrigerator until arsenic analysis. We also collected 89 biological fingernail samples from the individuals in the studied area. Forty fingernail samples were taken from participants living in the arsenic-contaminated villages, and the rest had no exposure to arsenic via drinking water. Then, the fingernail samples were cut using a stainless steel nail clipper and kept in plastic bags until measured.

### Measurement of arsenic in participant fingernail and groundwater samples

A simple digestion method with HNO_3_ and H_2_O_2_ was used to measure the concentration of arsenic in the fingernail samples. About 0.02–0.05 g of the fingernail sample was weighed and added to a 25-mL beaker containing 4 mL of HNO_3_ (65%) and 2 mL of H_2_O_2_ (30%). The beakers containing the samples are placed at 80–90 °C on a hot plate and heating was continued by adding a certain volume of HNO_3_ to change the color of the sample from dark brown to pale yellow. When the final volume of the sample reached 1 mL, the heating operation was stopped. After cooling the samples, 2–3 mL of distilled water was added to each sample and the prepared solution was passed through a Millipore filter and finally reached a volume of 5 mL. Finally, the concentration of arsenic in drinking water and the fingernail samples was determined by the ICP-OES apparatus^22,23^.

### Quality control and quality assurance (QC /QA)

Arsenic content in drinking water and fingernail samples were determined using an ICP-OES apparatus. HNO_3_ (65%) was of Suprapur^R^ grade and obtained from Merck Co (Germany). All the other reagents were purchased from Fluka (Sigma–Aldrich, Switzerland), and were of analytical grade. To achieve the precision of data, the arsenic levels in groundwater, fingernails, standard solutions (standard solution Fluka-51844, Sigma–Aldrich, Switzerland), and blank samples were examined in triplicate. In all repeated measurements, a relative standard deviation of 5–10% was achieved during the experiments^3,24^. Furthermore, to further verify the results, no human fingernail-certified reference material was available for comparison.

### Statistical analysis

In the present study, SPSS V.16.0 software was used to perform statistical calculations of the data with a significant level at *p* < 0.05. To check the normality of the data, the One-Sample Kolmogorov–Smirnov test was applied. Because the concentration of arsenic in fingernail samples was not normally distributed, non-parametric statistical tests such as Mann–Whitney U test were used to find significant differences between the groups of participants. The Chi-square test was applied to compare the distribution for the categorical variables such as BMI, gender, and smoking habits in the two reference and exposed groups. Also, an independent t-test was used to compare the age variable in the two reference and exposed groups. The association between arsenic concentration in groundwater and fingernail samples was evaluated using single and multiple linear regression models adjusted for age, BMI, gender, and smoking habits. Descriptive statistics including maximum, minimum, average, and standard deviation of arsenic concentration in fingernail samples were calculated using Excel 2013 Software (Microsoft Office).

### Consent to participate

All of the authors in this paper are acknowledged and listed as contributors, and they confirm the final version of the manuscript.

## Results

In the present study, the fingernail samples were used as a biomarker to assess human exposure to arsenic via drinking water consumption in individuals who lived in the arsenic-contaminated areas. It should be pointed that the individuals were allocated into the reference group (areas D, E, and F) with levels of 0.179 µg/L arsenic and the exposed group with different concentrations of arsenic in groundwater sources (region A = 200 µg/L, region B = 76.6 µg/L, and region C = 74.5 µg/L). To do this research, we collected 89 fingernail samples, of which 40 samples were from the participants living in arsenic-contaminated villages and the rest from those having no exposure to arsenic. Table 1 represents arsenic levels (μg/g) in the fingernail samples of the participants collected from exposed and reference villages of Kaboudrahang County. According to the results, the mean values of arsenic in fingernail samples of 1.78 μg/g with a range of 0.13–10.33 μg/g was observed in the exposed regions of the area studied compared to those from the reference areas (0.43 μg/g, with a range of 0.1–1.21 μg/g). This means that the average, minimum, and maximum concentrations of arsenic in the fingernail samples were higher in the participants living in arsenic-contaminated areas than in those living in areas without arsenic exposure. According to the results of the present study, in the villages exposed to arsenic via drinking water, 85% of the fingernail samples contained arsenic above the normal level, while in the villages without arsenic exposure, 28.57% of the fingernail samples had arsenic concentration higher than normal value.

**Table 1:** Summary statistics for arsenic concentrations (μg/g) in fingernail samples.

N	40
**Exposed area**	
Minimum	0.13
Maximum	10.33
STDEV.P	2.21
Average	1.78

N	49
**Reference area**	
Minimum	0.10
Maximum	1.21
STDEV.P	0.23
Average	0.43

*Normal level of arsenic in nail samples ranges from 20 to 500 μg/kg^22^.

The general properties of the participants studied, including age, gender, BMI, and smoking habits have been summarized in Table 2. As can be seen, the age average in the arsenic exposure group (34.8 ± 10.39 years) was significantly higher than that in the reference group (29.51 ± 10.8 years) (*p* = 0.022). Also, there was no significant difference between the two groups in terms of variables such as gender, smoking, and BMI (*p* ˃ 0.05). A significant difference was shown between arsenic levels in drinking water and the fingernail samples in two exposed and reference groups of the area (*p* < 0.001) (Table 2).

**Table 2:** The studied variables in the two exposed and referenced groups.

Variables	Exposed participants	Reference participants	*p-value*
Age (Mean ± SD)	34.8 ± 10.39	29.51 ± 10.8	0.022^a^
**BMI, N (%)**			
< 18.5	5 (12.5)	7 (14.4)	0.66^b^
18.5–24.9	21 (52.5)	21 (42.8)	
> 25	14 (35)	21 (42.8)	
**Gender, N (%)**			
Female	23 (57.5)	28 (57)	0.973^b^
Male	17 (42.5)	21 (43)	
**Smoking, N (%)**			
Yes	7 (17.5)	7 (14.3)	0.679^b^
No	33 (82.5)	42 (85.7)	
Arsenic in fingernail (Mean ± SD)	1.78 ± 2.21	0.43 ± 0.23	< 0.001^c^

SD standard deviation.

^a^Independent-samples T-test.

^b^Chi-square test.

^c^Mann–Whitney U test.

The results of the association between arsenic levels in drinking water sources and fingernail samples by using crude linear regression models have been depicted in Table 3. In the simple linear regression model, a significant relation was shown between the concentration of arsenic in fingernail samples with variables including gender (*p* = 0.019) and oral intake of arsenic through contaminated drinking water (*p* < 0.001). The results showed that females had significantly lower concentrations of arsenic in the fingernail samples (β = − 0.248, 95% CI − 0.843, − 0.078) compared with males, which is consistent with the results of other studies^25,26^. Likewise, a 1% increase of arsenic concentration in groundwater was associated with a 60% (β = 0.595, 95% CI 0.781, 1.413) increase in the fingernail samples arsenic. Although no significant relation was found between fingernail arsenic and variables such as age and smoking habits, the results indicated that arsenic concentration in fingernail samples increased with age (β = 0.199, 95% CI 0.00, 0.035, *p* = 0.062) and smoking (β = 0.192, 95% CI − 0.043, 1.011, *p* = 0.071). The results also showed that obese participants had lower fingernail arsenic compared to those with a normal weight (β = − 0.125, 95% CI − 0.069, 0.018, *p* = 0.242).

**Table 3:** Variables associated with the concentration of arsenic in fingernails.

Crude					Adjusted^a^				
Variables	β	95% CI		*p-value*	Variables	β	95% CI		*p-value*
		Lower	Upper				Lower	Upper	
Age	0.199	0.00	0.035	0.062	Age	0.152	− 0.007	0.033	0.2
BMI	− 0.125	− 0.069	0.018	0.242	BMI	− 0.152	− 0.077	0.015	0.184
Gender	− 0.248	− 0.843	− 0.078	0.019*	Gender	− 0.208	− 0.748	− 0.023	0.037*
Smoking	0.192	− 0.043	1.011	0.071	Smoking	0.002	− 0.513	0.525	0.982
Exposure to arsenic	0.595	0.781	1.413	< 0.001*	Exposure to arsenic	0.558	0.709	1.348	< 0.001*

*CI* confidence interval.

^a^Adjusted for age, gender, BMI, smoking and exposure to arsenic via drinking water.

*p-value < 0.05.

In the multivariate linear regression model adjusted for age, gender, BMI, smoking, and exposure to arsenic, the variables including gender and arsenic levels in drinking water were significantly associated with fingernail sample arsenic (*p* < 0.05). Moreover, in this stage, a 1% increase in groundwater arsenic was associated with a 56% ((β = 0.558) increase of arsenic in the fingernail samples. The arsenic concentration in the fingernail samples of the female participants was lower than those in male participants (β = − 0.208). Also, no statistically significant relationship was found between arsenic concentration in the fingernail samples and variables like age, BMI, and smoking. However, similar to the findings achieved in simple linear regression, with increasing age (β = 0.152), smoking (β = 0.002), and decreasing BMI (β = − 0.152), the arsenic concentration of the fingernail samples increased.

## Discussion

In recent decades, the study on biological monitoring in epidemiological and toxicological research to assess the risk of human exposure to pollutants from different routes has been of great importance. In this regard, due to the widespread occurrence of arsenic in the environment and drinking water sources, as well as its potential for adverse effects on human health, biological monitoring is used as a valuable tool to assess exposure to arsenic through natural and anthropogenic pathways^15,16^.

The main source of groundwater contamination with arsenic could be attributed to the geological structure of the study area, which comprised limestone, Jurassic sandstone, marl, metamorphic rocks, and shale. Also, most portions of the area belong to the northwestern part of the Sanandaj-Sirjan zone and the southwestern part of the Zagros thrust belt^27,28^. Another reason for arsenic contamination in drinking water sources is the proximity of the area to Kurdistan Province, where the presence of arsenic in drinking water sources has been reported in some areas^29,30^. According to the results of the present study, the average, minimum, and maximum concentrations of arsenic in the fingernail samples were higher in the participants living in arsenic-contaminated areas than in those living in areas without arsenic exposure.

Spatial variations and regional distribution of arsenic contents in toenail samples and drinking water supplies of Nova Scotia in Canada were examined by Dummer et al. They also assessed the geological and environmental characteristics related to high concentrations of arsenic in drinking water sources. Based on the findings of this study, the levels of arsenic in drinking water samples where less than the detection limit of the method (478 µg/L). In their research, they stated that the contamination of drinking water sources with arsenic originated from the geological structure of the study area. Also, in this research close associations were observed between the high levels of arsenic in toenail samples and drinking water supplies, which is consistent with the findings of our study^31^.

According to the results, in the villages exposed to arsenic, 85% of the fingernail samples contained arsenic above the normal level, while in the villages without arsenic exposure, 28.57% of the fingernail samples had arsenic concentration higher than normal value.

The observations of a study by Chakraborti et al., who analyzed the concentration of arsenic in 176 biological samples (hair, urine, and nail), indicated that 69 participants had skin lesions caused by arsenic exposure, and the rest did not have arsenical skin lesions. In this study, normal arsenic levels in hair, nail, and urine samples were reported to be 20–200 µg/kg, 20–500 µg/kg, and < 100 µg/L, respectively. They found that 100% of the biological samples had an arsenic concentration above normal values^22^.

In the multivariate linear regression model adjusted for age, gender, BMI, smoking, and exposure to arsenic, the variables including gender and arsenic levels in drinking water were significantly associated with fingernail sample arsenic (*p* < 0.05).

In a study by Schmitt et al., human nail samples were used as biomarkers for arsenic exposure in the Mongolia region, China. In this study, the nail samples were collected from 32 participants. A total of 19 individuals were exposed to high concentrations of arsenic through drinking water (264–648 µg/L), and 13 subjects were exposed to low concentrations of arsenic (0.3–9.8 µg/L), representing as the control group. A significant relation was found between arsenic levels in drinking water and the nail samples in this study. Also, based on the findings of this study, the concentration of arsenic in the nail samples in males due to greater water consumption was higher than in the female participants, which is consistent with the findings of the present study^32^.

In another study, the correlation between arsenic concentrations in drinking water sources and toenail samples in Nova Scotians was investigated by Yu et al. A total of 960 "samples of male and female" aged 35- 69 years were selected for arsenic analysis in toenail clipping samples. In this study, information including age, gender, drinking water source, treatment use, ethnicity, level of education, employment status, household income, smoking status, physical activity, and BMI was asked through a questionnaire from all the participants. Based on the findings of their study, a statistically significant association was observed between the arsenic concentration of drinking water sources and toenail samples (r = 0.46, *p* < 0.0001), which is consistent with the findings of our study. The results of this study also showed that the level of arsenic in the toenail samples in female (β = − 0.132, 95% CI − 0.238, − 0.026, *p-value* = 0.0268), obese individuals (β = -0.263, 95% CI − 0.386, − 0.141, *p* < 0.0001) and participants with higher levels of family income (β = − 0.152, 95% CI − 0.292, − 0.011, *p* = 0.0345) were significantly lower than others^33^.

In a study conducted by Liu et al., the association between arsenic uptake and human health risk was evaluated using biomarkers including hair, nails, urine, and saliva. The results of their investigation represented that the concentration of arsenic in biological samples was higher in males, older, and participants with skin lesions than those in others. Also, they reported that females had a higher methylation capacity than males, so the concentration of arsenic in biological samples was lower in females, which is consistent with the findings of the present study^26^.

The present study results showed that the use of the fingernail samples as a biomarker to evaluate arsenic exposure through ingestion of drinking water was appropriate. Nail samples contain high amounts of scleroproteins (like keratin) and sulfhydryl groups; it should be pointed out that arsenic has a strong affinity for binding to these sulfhydryl groups. Also, due to the feeding of germinal nail matrix from a rich blood source, arsenic deposition in nail samples occurs shortly after consumption. It can be concluded that because of the slow growth rate, less probability of external pollution compared to other biomarkers (like hair samples), easier collection, and non-invasive properties, the use of nail samples (fingernail and toenail samples) as a suitable biomarker to study exposure to environmental pollutants is on the rise^18,32^. It should be noted that the levels of measured arsenic in nail samples in different studies are reported in Table 4.

**Table 4:** The concentration of arsenic in nail samples collected from different studies.

Arsenic in nail samples	Range	References
Ganga plain, India	1254–20,202 µg/kg	^22^
Hetao Basin, Inner Mongolia	84–1290 µg/kg	^26^
Shahpur block, Bihar state, India	469–36,520 µg/kg	^34^
Nadia district, West Bengal, India	80–36,840 µg/kg	^23^
Middle and Lower Ganga plain	6.1–24 µg/g	^35^
Majuli, Assam, India	426–11,725 µg/kg	^36^
Present study	0.13–10.33 µg/g	–

## Conclusion

The current study aimed to use fingernail samples as a human biomarker for arsenic exposure via the consumption of contaminated groundwater. Our results indicated that geological structures have an important role in the occurrence of higher values of arsenic in groundwater sources and fingernails. Also, this research displayed that the contents of fingernail arsenic in 50 and 4.08% of the samples collected from arsenic-contaminated and reference areas were higher than the normal arsenic values of nails (0.43–1.08 µg/g), respectively. Based on the results of adjusted multiple linear regression, the concentration of arsenic in the fingernails of the participants living in the arsenic-contaminated areas was significantly higher than that in the individuals in the reference areas (*p* < 0.001). Moreover, a statistically significant association was shown between arsenic in the fingernail samples and gender; females had significantly lower values of arsenic in fingernails than males (*p* = 0.037). Fingernails arsenic contents were not significantly affected by other variables including age, smoking habits, and BMI (*p* > 0.05). It was found that Based on the overview of the results achieved in this work, more research with a higher number of participants are recommended to improve the benefits of such studies and their application in the medical sciences.

### Study limitations

There are several major limitations in the current study that should be noted. First, due to financial constraints, the number of fingernail samples analyzed was small, thereby reducing the accuracy of our analysis. Second, we did not investigate the relationship between arsenic concentrations in drinking water sources and other biomarkers such as hair, urine, blood, and agricultural products. Third, we only measured the values of total arsenic in fingernail samples and did not specify the values of organic and inorganic arsenic separately. The present study attempted to investigate the relationship between arsenic concentrations in drinking water sources and fingernail samples as biomarkers, but future studies with more samples size are required to investigate the relation of arsenic bioaccumulation (accumulation in hair, urine, saliva, and blood samples), diet and health.

## Data Availability

All of the data have been reported in the main manuscript body.
